# Mechanistic insights into proton-coupled substrate translocation of nucleoside proton symporters

**DOI:** 10.1016/j.jbc.2025.108357

**Published:** 2025-02-25

**Authors:** Qingjie Xiao, Xinyi Chen, Chen Wang, Yingying He, Dong Deng, Bo Sun

**Affiliations:** 1 Shanghai Advanced Research Institute, Chinese Academy of Sciences, Shanghai, China; 2 Department of Obstetrics, Key Laboratory of Birth Defects and Related Disease of Women and Children of MOE, State Key Laboratory of Biotherapy, West China Second Hospital, Sichuan University, Chengdu, China; 3 NHC Key Laboratory of Chronobiology, Sichuan University, Chengdu, China

**Keywords:** nucleoside transport, proton-coupled translocation, MFS, GXXXD motif, conformational change

## Abstract

The nucleoside proton symporter (NHS) family proteins are part of the major facilitator superfamily and are responsible for transporting nucleosides from the extracellular environment into the cell. Structural and biochemical analysis of NupG, a prototypical NHS member, have pinpointed the critical residues involved in substrate binding. However, the proton-coupled mechanism diving substrate translocation in NHS proteins has remained elusive. In previous research, we identified Asp323 in NupG as a potential protonation site. In this study, using X-ray crystallography, molecular dynamics simulations, and biochemical assays, we discovered that the deprotonation of Asp323 in NupG, or the equivalent Asp315 in YegT, (another NHS family member) triggers a local conformational change in the TM10 region of NHS transporters. Notably, this protonation site is part of a novel motif (GXXXD) located in the middle of the TM10 transmembrane helix in NHS proteins. Further biochemical studies suggest that this local conformational change in the GXXXD motif plays a role in coordinating substrate release, ultimately facilitating substrate translocation. Our findings provide valuable insights into the molecular mechanism of nucleoside transport and expand the understanding of the diverse transport mechanisms within the major facilitator superfamily.

The major facilitator superfamily (MFS), the largest family of secondary transporters, is essential for regulating solute transport across biomembranes. These proteins typically consist of 12 transmembrane helices arranged into two transmembrane domains connected by an internal loop (1, 2, 3). After decades of research, significant progress has been made in understanding the transport mechanism of MFS family proteins (1, 2, 3).

MFS members are categorized as uniporters, symporters, and antiporters based on their transport mechanisms (1, 2, 3). Notably, investigating the proton or sodium ion coupling in symporters and antiporters has long been a challenging area in transporter research. However, recent studies have provided significant insights into the coupling mechanisms of key MFS transporters, including LacY (4, 5, 6) and XylE (4, 7, 8). Using LacY as an example, researchers have proposed that two protonation sites influence substrate binding and induce conformational changes at different states (4). In the case of XylE, Asp27 in TM1 serves as the protonation site. The protonation or deprotonation of Asp27 may regulate XylE’s conformational transitions (4, 7, 8). Furthermore, other transporter families, such as the neurotransmitter-sodium symporter family and the concentrative nucleoside transporter family, also demonstrate sodium ion or proton coupling mechanisms (9, 10). The transporter binds a proton or sodium ion to form the substrate-binding pocket, enabling substrate recognition. However, the direct connection between the protonation states of symporters and substrate release remains unexplored in current research.

In previous work, we determined the structure of NupG, a prototype transporter from the nucleoside proton symporter (NHS) family, and identified a crucial protonation site at Asp323 (11). We observed that the protonation state of Asp323 significantly influences substrate binding, yet the link between Asp323's protonation and substrate translocation remains unknown. To investigate whether this protonation site is a common feature within the NHS subfamily, we conducted a structural analysis of another NHS subfamily protein, YegT (12). Interestingly, we discovered that the Asp315 residue in YegT, analogous to Asp323 in NupG's, is also protonated. This suggests that protonation might have a universal role in regulating substrate binding and release, though the specific mechanisms are still unclear. To further explore this, we used a combination of crystallography, biochemical assays, and molecular dynamics (MD) simulations to study the interactions between protonation states and substrate release. Our research focuses on gaining a deeper understanding of the transport mechanisms of MFS family proteins in their protonated state, offering fresh insights into this area of study.

By conducting sequence alignment of NHS family members, we identified a critical residue within the conserved GXXXD motif. Unlike the previously studied GXXXD motifs located on the cytoplasmic L2-L3 or L8-L9 loops, this motif is situated within TM10 in our study. We performed 200-nanosecond-long MD simulations on YegT and NupG, which revealed that the protonation of the D residue within the GXXXD motif plays a vital role in maintaining the interaction between TM10 and TM7, a key factor for substrate binding. Moreover, our simulations demonstrated that the proton from D323 can be transferred to E264. Subsequent isothermal titration calorimetry (ITC) experiments confirmed that the protonation state of E264 influences NupG’s substrate binding affinity. Based on these findings, we propose a proton-coupling mechanism for NHS subfamily proteins, shedding light on the process of substrate release. This research contributes a novel perspective for understanding the transport mechanisms of MFS family proteins.

## Results

### The conserved GxxxD motif of NHS transporters

**Figure 1:**
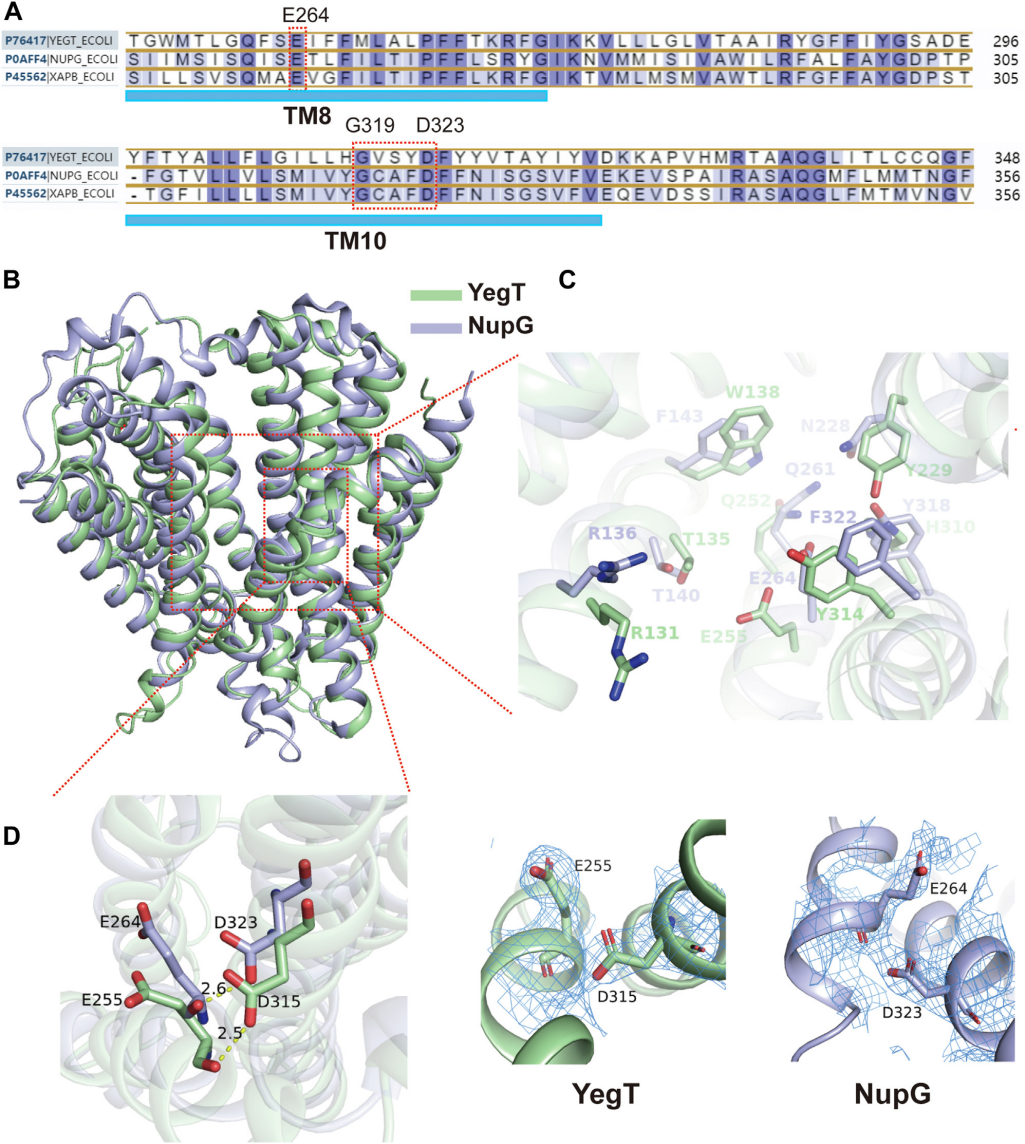
**Comparative structure of the YegT and NupG proteins**. *A*, the sequence of the GxxxD motif that is found in the TM10 of NHS transporters. *B*, a comparative overview of the structure of the YegT and NupG proteins. *C*, detailed perspective of the substrate-binding cavities in the YegT and NupG proteins. *D*, detailed perspective of the protonation sites in the YegT and NupG proteins. The PDB accession number for the NupG protein is 7DLG. The 2Fo-Fc density map was contoured at 3.0 σ and as the *blue mesh*. PDB, Protein Data Bank; NHS, nucleoside proton symporter.

Typically, MFS transporters contain two conserved motifs: motif-A (GxxxD) and motif-B (RxxqG) (2, 13). As a MFS transporter, NupG also possesses a motif-A, located at residues 319 to 323 (Fig. 1*A*). Notably, NupG’s motif-A is positioned in the middle of TM10, whereas in classical MFS transporters, motif-As is generally found in the loop between TM2 and TM3 (2). Furthermore, NupG’s motif-A lacks the subsequent unique "RxGRR" sequence (2). Sequence alignment of three NHS family members revealed that this distinctive motif-A is conserved within the NHS family (Fig. 1*A*).

To validate this observation, we solved the crystal structure of YegT, another member of the NHS family. We introduced a point mutation in YegT (YegT_K267A_) to improve the quality of the crystal and crystallized the protein using the lipidic cubic phase method. Through molecular replacement, we determined the structure of YegT_K267A_ at a resolution of 3.1 Å (Fig. S1). The asymmetric unit, contained two YegT molecules, designated as molecule A and molecule B. Notably, both molecules were captured in nearly identical conformations, with a RMSD of 0.17 Å across 332Cα atoms. Consequently, only molecule A was used for further structural analysis.

YegT_K267A_ displays the characteristics MFS fold, consisting of 12 transmembrane helices (TM1-12). The N-terminal domain (TM1-6) and the C-terminal domain (TM7-12), connected by a loop, form an inward-facing cavity between them (Fig. S1). This indicates that YegT_K267A_ was captured in an inward-facing conformation (Fig. S1). Notably, Lys267 is located in the loop connecting TM8 and TM9 (Fig. S1), which is distant from the substrate-binding site. This placement ensures that the mutation has minimal effect on the substrate-binding region.

A structural comparison between two NHS members, NupG and YegT, revealed that both proteins share similar structural features, with an RMSD of approximately 1.57 Å across 316 Cα atoms, despite having only about 27% sequence identity (Fig. 1*B*). Further analysis of the substrate-binding cavities in YegT and NupG highlighted differences in the residues within these cavities. Specifically, residues Phe143, Try318, Phe322, and Asn228 in NupG, which are closely associated with uridine binding, are not conserved at the corresponding positions in YegT (Fig. 1*C*). This suggests that the nucleoside bound by YegT may differ from that of NupG. Previous studies have shown that NupG binds a range of nucleosides, including uridine, adenosine, cytidine, and guanosine, as detected by the ITC method (11). However, attempts to determine YegT’s affinity for these nucleosides using ITC did not produce significant results. This suggests that there are significant differences between the two proteins in substrate binding. Interestingly, the conserved motif-As of YegT and NupG are located at the same position in the middle of TM10. The protonation site, Asp315 in YegT, and the corresponding residue Asp323 in NupG, are situated within this conserved motif. The pKa value of Asp315 in YegT, calculated using the PROPKA3 software (https://github.com/jensengroup/propka) (14), is 7.96. Given that the measured pH obtained for the crystallization condition was 5.8, this strongly suggests that Asp315 is protonated. Notably, the side-chain carbonyl oxygen atom of Asp315 is located solely 2.5 Å apart of the main-chain carbonyl oxygen atom of Glu255. This further indicates that Asp315 is protonated, as illustrated in Figure 1*D*. Similarly, in the previously resolved structure of the NupG protein, the side-chain carbonyl oxygen atom of Asp323 forms a hydrogen bond with the main-chain carbonyl oxygen atom of Glu264, with both atoms located at a distance of only 2.6 Å (Fig. 1*D*). To confirm that the protonation state of Asp323 does actually influence substrate affinity in the NupG protein, we generated a NupG_D323A_ mutant and conducted a nucleoside binding assay (11).

Interestingly, the NupG_D323A_ mutant exhibited similar uridine binding affinity at both pH 6.0 and 8.0, whereas the WT NupG protein showed a significant variation in substrate affinity depending on the pH (11). This suggests that the substrate binding of the NupG protein is regulated by the protonation and deprotonation states of Asp323. Given the high structural similarity and the identical protonated residues that can be found in both YegT and NupG proteins, we hypothesize that NHS-family transporters likely share similar protonation mechanisms to regulate substrate translocation.

### MD simulation of NHS transporters

**Figure 2:**
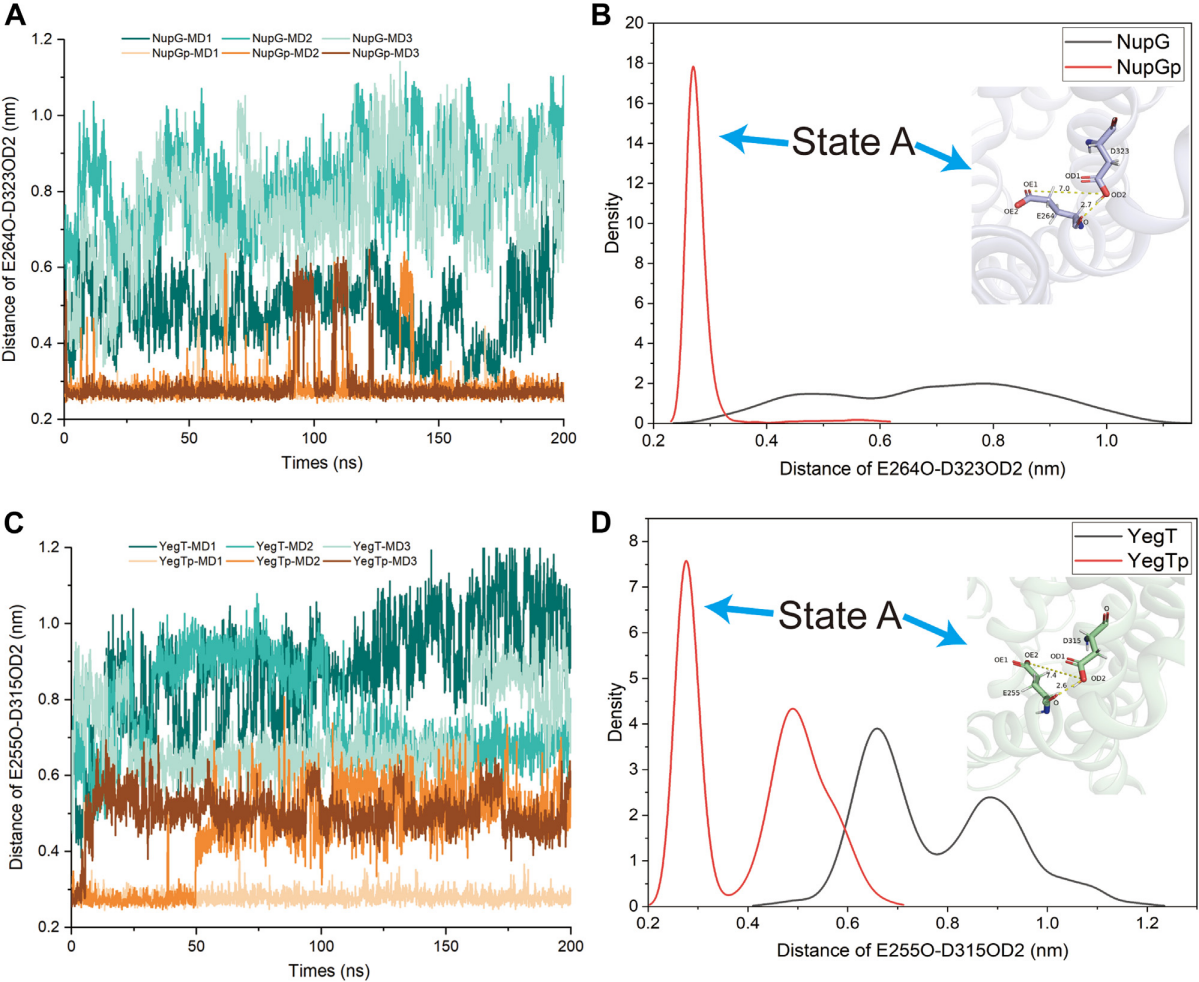
**Simulations of the molecular dynamics of the D323 and D315 sites in the NupG and YegT proteins at both protonated and deprotonated states**. *A*, distance variation across a 200-nanosecond interval between the carbonyl oxygen of the D323 site (TM10) and the main chain carbonyl oxygen of the E264 site (TM8) in the NupG protein. *B*, statistical presentation of the density distribution of the distance between the carbonyl oxygen of the D323 site (TM10) and the main chain carbonyl oxygen of the E264 site (TM8) in the NupG protein across a 200-nanosecond interval. *C*, distance variation across a 200-nanosecond interval between the carbonyl oxygen of the D315 site in the YegT protein and the main chain carbonyl oxygen of the E255 site. *D*, statistical presentation of the density distribution of the distance between the carbonyl oxygen of the D315 site (TM10) and the main chain carbonyl oxygen of the E255 site (TM8) in the YegT protein across a 200-nanosecond interval. NupG-MD1, NupG-MD2, and NupG-MD3 refer to three independent simulations of NupG in a deprotonated state. NupGp-MD1, NupGp-MD2, and NupGp-MD3 refer to three independent simulations of NupG in a protonated state.

To investigate how the protonation states of key residues in NHS transporters (Asp323 in NupG and Asp315 in YegT) affect substrate binding, we conducted 200 ns equilibrium simulations for both NupG and YegT (Fig. S2). The structural models of WT NupG and YegT were utilized in MD simulations. Our investigation centered on the behavior of Asp323 and its associated residue Glu264, driven by an interest in the relationship between Asp323’s protonation state and substrate-binding in NupG. By examining the distance between E264O and D323OD2, we observed that the protonated side chain of Asp323 consistently maintained an average distance of approximately 3 Å from the main chain oxygen of Glu264 (State A), suggesting the formation of a hydrogen bond between Asp323 and Glu264 (Fig. 2, *A* and *B*).

This result suggests that the protonation state of Asp323 is crucial for maintaining the interaction between the side chain of Asp323 and the main chain oxygen of Glu264. In contrast, when Asp323 is deprotonated, the distance between its side chain and the main chain oxygen of Glu264 typically exceeds 4 Å (Fig. 2, *A* and *B*). Theoretically, deprotonated Asp323 cannot donate a proton to facilitate hydrogen bond formation with the main chain oxygen of Glu264. Instead, the side chain might create steric hindrance. Additionally, the local conformations of these two negatively charged residues adjust due to electrostatic repulsion, directly influencing the conformations of TM10 and TM8. Consequently, this subtle change is likely to impact substrate binding, as several residues from TM10 and TM8 are involved in forming extensive polar contacts with the nucleoside.

**Figure 3:**
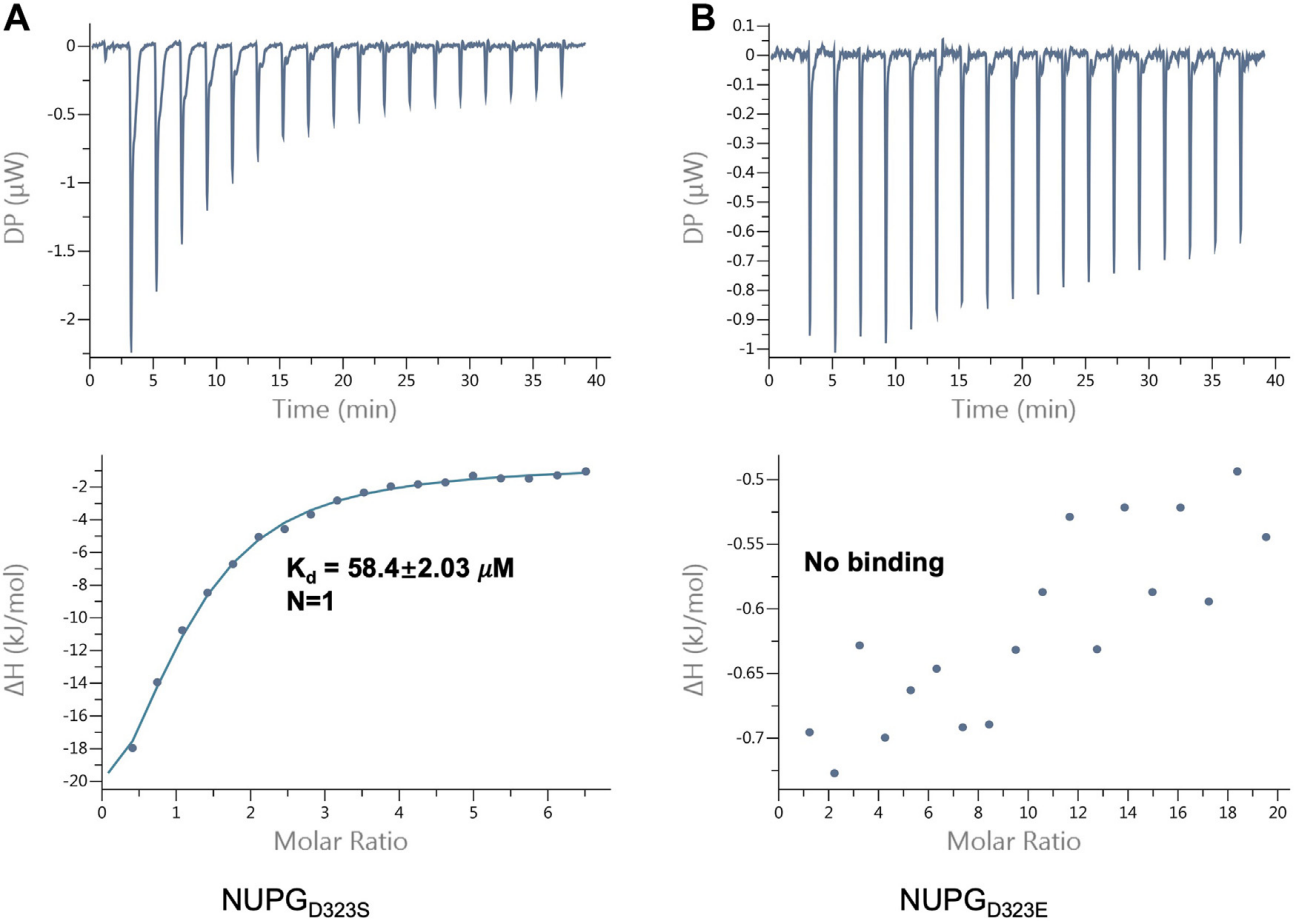
**Measuring the affinity between the D323S and D323E mutants using uridine**. *A*, the binding affinity between the NupG_D323S_ mutant protein and the uridine nucleoside. *B*, the binding affinity between the NupG_D323E_ mutant protein and the uridine nucleoside.

Previously, we identified the NupG_D323A_ variant, which exhibited enhanced uridine-binding (11). The shorter side chain of alanine likely avoids steric hindrance with Glu264. To further confirm that steric hindrance from the Asp323 residue affects substrate binding, we constructed NupG_D323S_ variants and measured their affinities for uridine. Both variants demonstrated enhanced substrate binding, with NupG_D323S_ showing a binding affinity of 58.4 ± 2.03 μM, similar to that of NupG_D323A_. However, no detectable binding was observed between NupG_D323E_ and uridine (Fig. 3). These biochemical findings support the notion that local conformational changes involving Asp323 can influence substrate binding. Additionally, the observed distance between Asp315 (TM10) and Glu255 (TM8) in YegT is comparable to that in NupG, suggesting a conserved mechanism among NHS transporters (Fig. 2, *C* and *D*).

**Figure 4:**
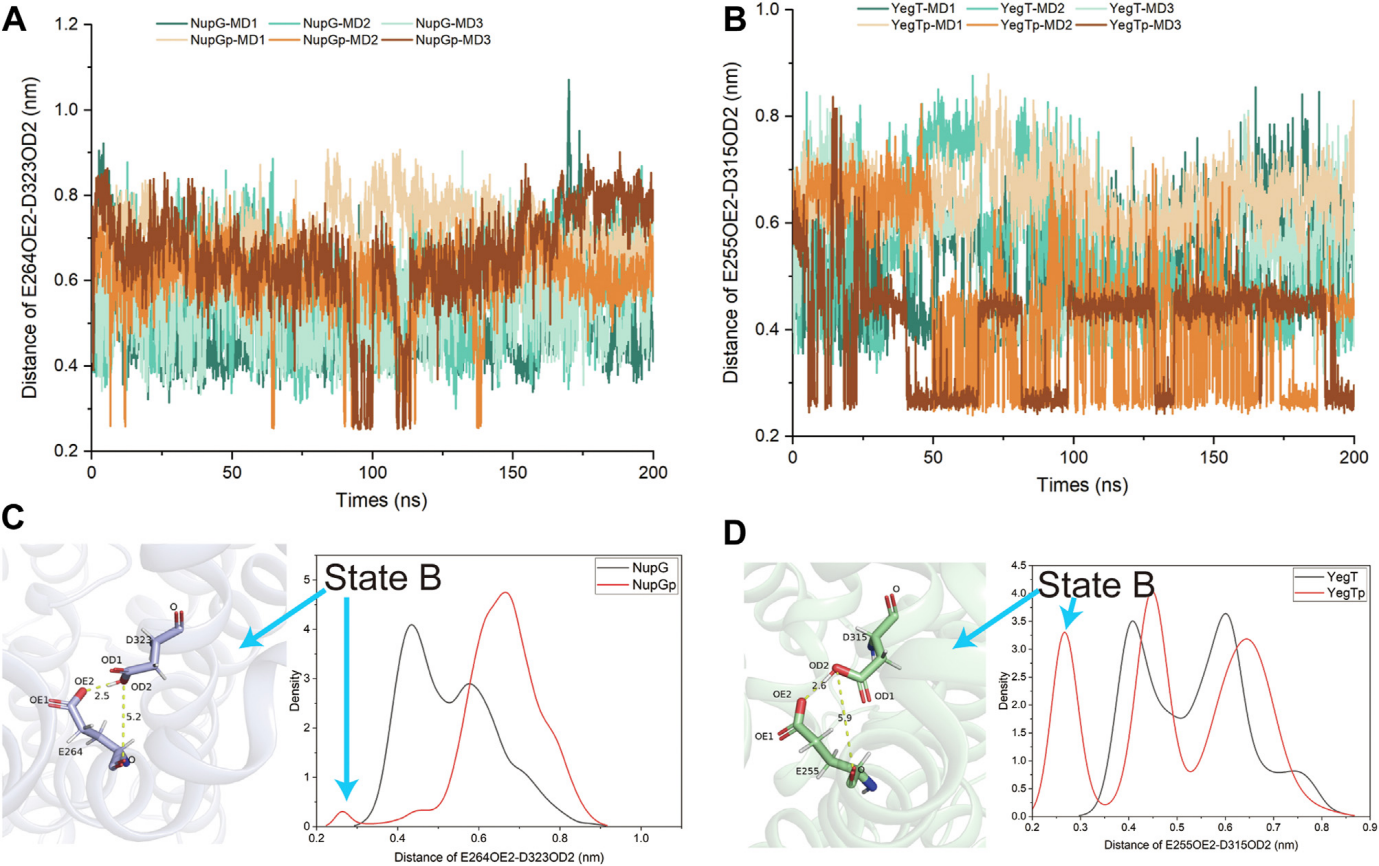
**Simulations of the molecular dynamics of the D323 and D315 sites in the NupG and the YEGT proteins at both protonated and unprotonated states**. *A*, distance variation across a 200-nanosecond interval between the carbonyl oxygen on the side chain of the D323 site and the carbonyl oxygen on the side chain of the E264 site in the NupG protein. *B*, statistical presentation of the density distribution of the distance between the carbonyl oxygen on the side chain of the D323 site (TM10) and the side chain oxygen atom of the E264 site (TM8) in the NupG protein over across a 200-nanosecond interval. *C*, distance variation across a 200-nanosecond interval between the carbonyl oxygen on the side chain of the D315 site and the carbonyl oxygen on the side chain of the E255 site in YegT protein. *D*, statistical presentation of the density distribution of the distance between the carbonyl oxygen on the side chain of the D315 site (TM10) and the side chain oxygen atom of the E255 (TM8) in the YegT protein across a 200-nanosecond interval.

**Figure 5:**
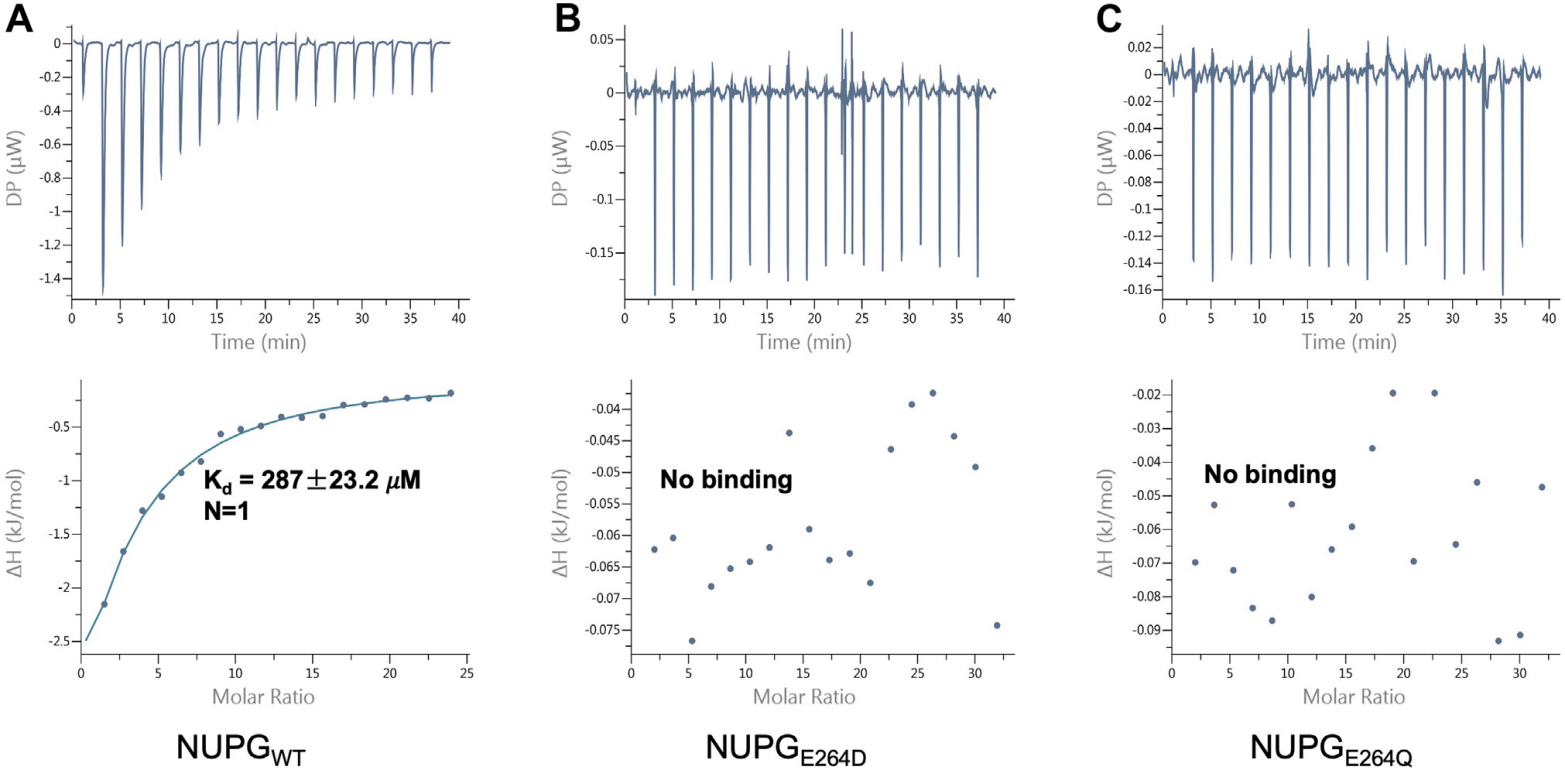
**ITC analysis of the affinity between several NupG mutants and the uridine nucleoside. Binding affinities were measured using ITC**. *A*, the binding affinity between the NupG protein and the uridine nucleoside. *B*, the binding affinity between the NupG_E264D_ mutant protein and the uridine nucleoside. *C*, the binding affinity between the NupG_E264Q_ mutant protein and the uridine nucleoside. ITC, isothermal titration calorimetry.

Interestingly, another distribution was observed in the distance between the side chains of Asp323 in NupG and Asp315 in YegT and the oxygen atoms on the side chains of Glu264 in NupG (Fig. 4, *A* and *C*) and Glu255 in YegT (Fig. 4, *B* and *D*), around 3 Å. Although this state (referred to as state B) was infrequent, it suggests the potential for proton transfer from Asp323 (NupG) or Asp315 (YegT) to Glu264 (NupG) or Glu255 (YegT). To determine whether the substrate binding affinity of NupG is significantly reduced upon the protonation of Glu264 (NupG)/Glu255 (YegT), we mutated Glu264 to glutamine (Q) to mimic its protonated state, as glutamine retains a similar size, can form hydrogen bonds, and mimics the neutral charge of protonated glutamic acid. The uridine binding of NupG_E264Q_ showed a substantial decrease (Fig. 5*B*). These findings indicate that after proton transfer to Glu264, the substrate is more likely to be released from the NupG cavity in the deprotonated state.

**Figure 6:**
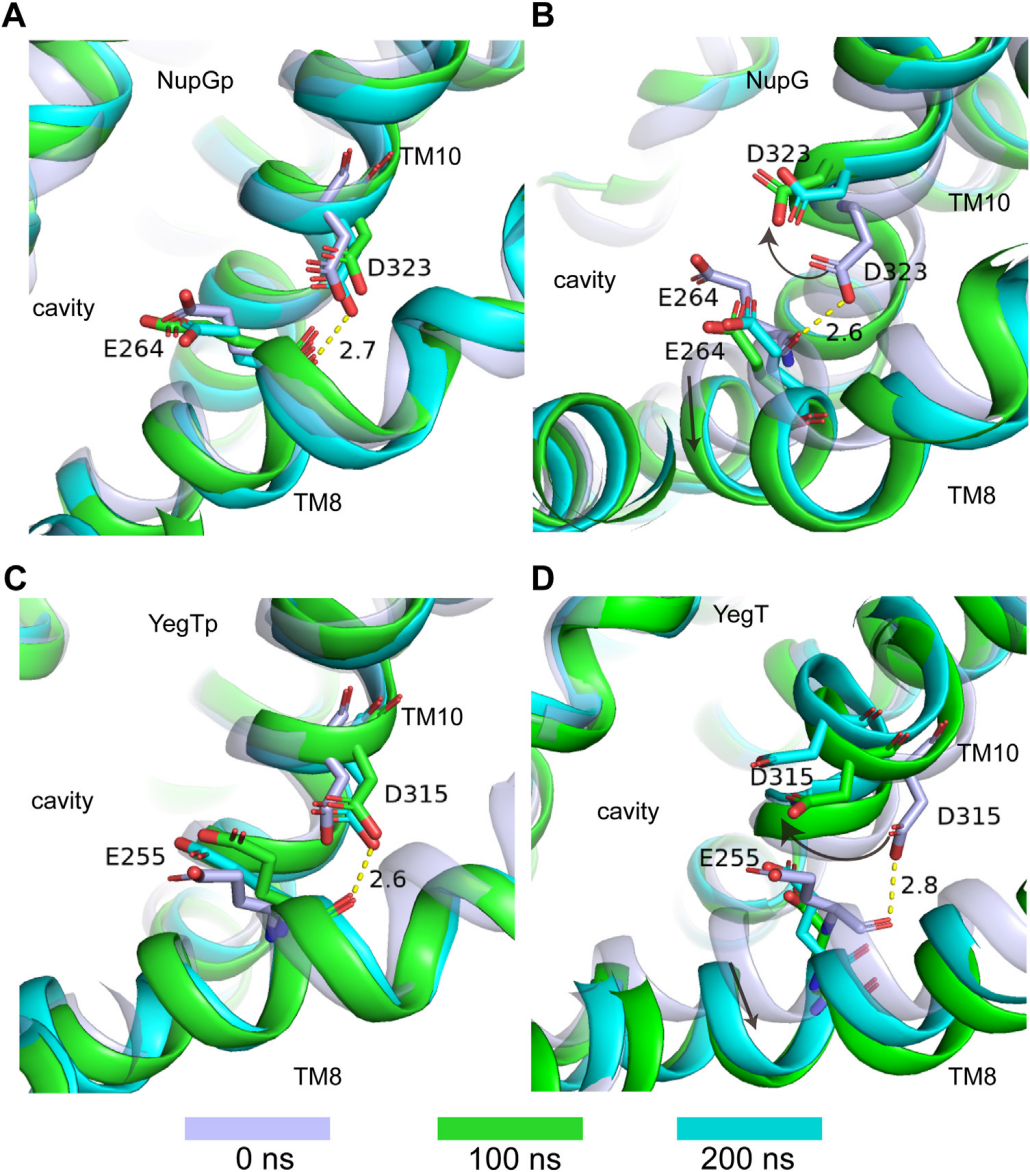
**Various snapshots depicting local conformational changes of the NupG and YegT proteins at both protonated and deprotonated states.** The *blue-gray* color represents the initial structure, *green* indicates the structure at 100-nanoseconds, and *cyan* denotes the structure at 200-nanoseconds. *A*, the protonated state of the NupG protein, showing the local conformational changes at the potential protonation site. *B*, the deprotonated state of the NupG protein, showing the local conformational changes at the potential protonation site. *C*, the protonated state of the YegT protein, showing the local conformational changes at the potential protonation site. *D*, the deprotonated state of the YegT protein, showing the local conformational changes at the potential protonation sites.

**Figure 7:**
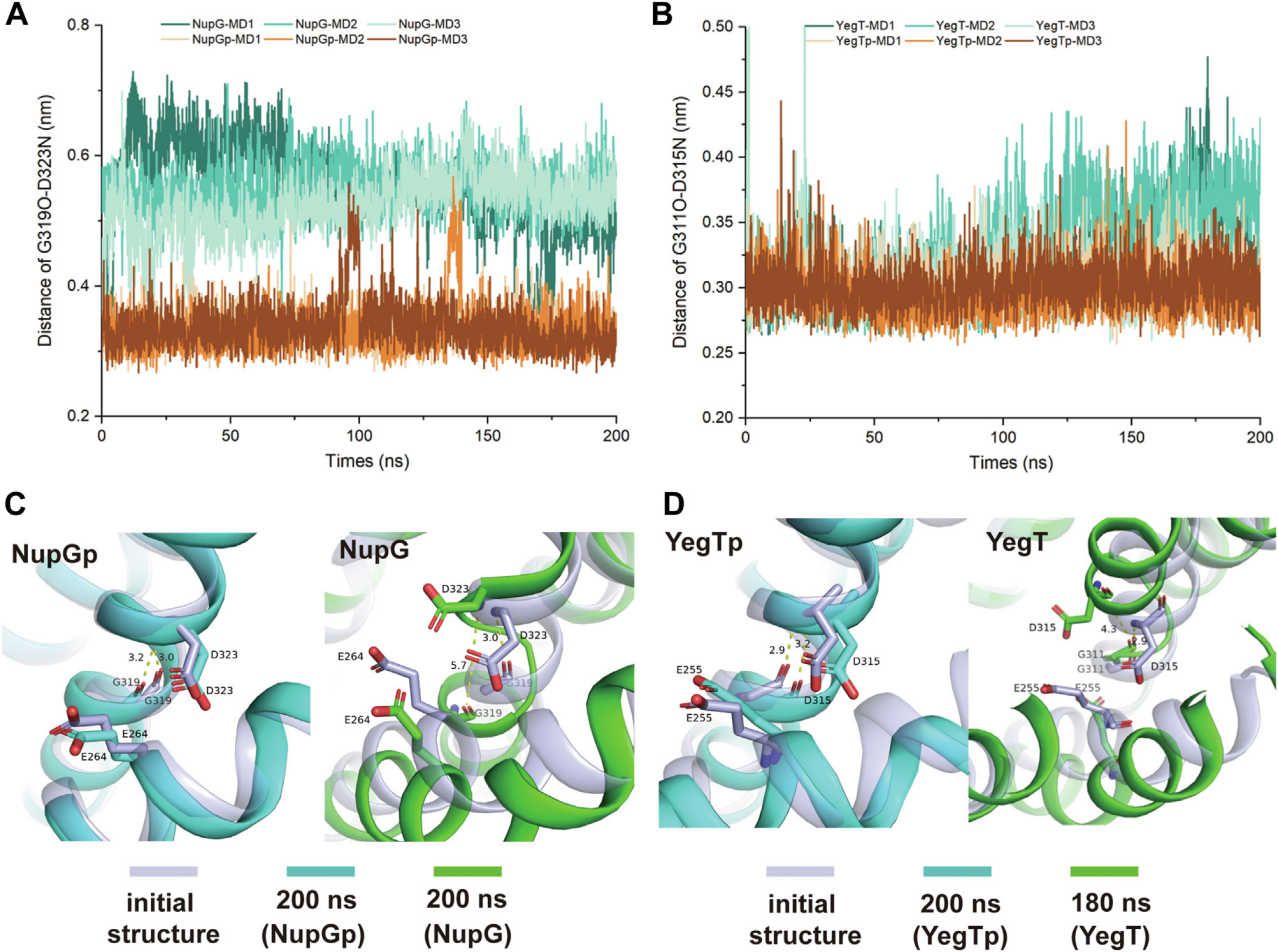
**Changes in the distance between the carbonyl oxygen atom of the G residue and the nitrogen atom of the D residue within the GXXXD motif**. *A*, changes in the distance between the main chain carbonyl oxygen atom of the G residue and the main chain nitrogen atom of the D residue within the GXXXD Motif in the NupG protein. *B*, changes in the distance between the main chain carbonyl oxygen atom of G and the main chain nitrogen atom of D within the GXXXD motif in the YegT protein. *C*, snapshot images of the NupG protein obtained at different time points. *D*, snapshot images of the YegT protein obtained at different time points.

Local conformational changes are essential in the deprotonated state to prevent steric hindrance. MD simulation results indicated that in the deprotonated state, the Asp323 residue of NupG and the Asp315 of YegT undergoes an approximately 90-degree rotation, accompanied by a noticeable outward shift of TM8. Snapshots taken at the start of the simulation, as well as at 100 ns and 200 ns, visually captured this change (Fig. 6). Based on these structural observations, we hypothesized that the outward shift of TM8 would directly impact the position of residue Glu264 in NupG or Glu255 in YegT. To test whether the shift in Glu264 of NupG leads to the loss of nucleoside-binding capability, we generated the NupG_E264D_ variant to mimic this residue shift. Notably, ITC results showed that the binding affinities between NupG_E264D_ and the substrate were undetectable at pH 6.0 (Fig. 5*C*). Consequently, these local conformational changes may lead to a loss of substrate affinity. Additionally, MD simulations revealed that the α-helix formed by the protonation site Asp and the fourth upstream residue Gly becomes prone to destabilization in the deprotonated state. Sequence alignment of TM10 from various NHS members (Fig. S3) showed that glycine and aspartic acid are highly conserved, forming the GXXXD motif, which constitutes a helical turn. In a typical α-helix, the amide nitrogen (N-H) of one amino acid forms a hydrogen bond with the carbonyl oxygen (C=O) of another amino acid three residues away, with oxygen and nitrogen atoms are approximately 3 Å apart. To illustrate the disruption of this helix more clearly, we analyzed the distances between the carbonyl oxygen atoms of Gly319 (NupG) and Gly311 (YegT) and the main chain nitrogen atoms of Asp323 (NupG) and Asp315 (YegT) over 200 ns (Fig. 7, *A* and *B*). In the deprotonated state, the distance between the carbonyl oxygen atom on glycine and the main chain nitrogen atom of aspartic acid increased, sometimes even exceeding 4 Å. Interestingly, this feature is more pronounced in the NupG protein (Fig. 7, *A* and *B*).

Furthermore, snapshots taken at various time points revealed significant helix disruption within TM10 (Fig. 7, *C* and *D*).

These observations strongly suggest the TM10 helix has a decreased stability under deprotonated conditions. However, previous studies indicated that glycine residues are able to disruptα-helix formation, with the GXXXD motif predominantly found in the loop regions of the MFS protein family. Based on this analysis, we hypothesize that the GXXXD motif plays a role in modulating the stability of the TM10 helix across different protonation states, thereby further influencing the binding and release of different substrates.

## Discussion

**Figure 8:**
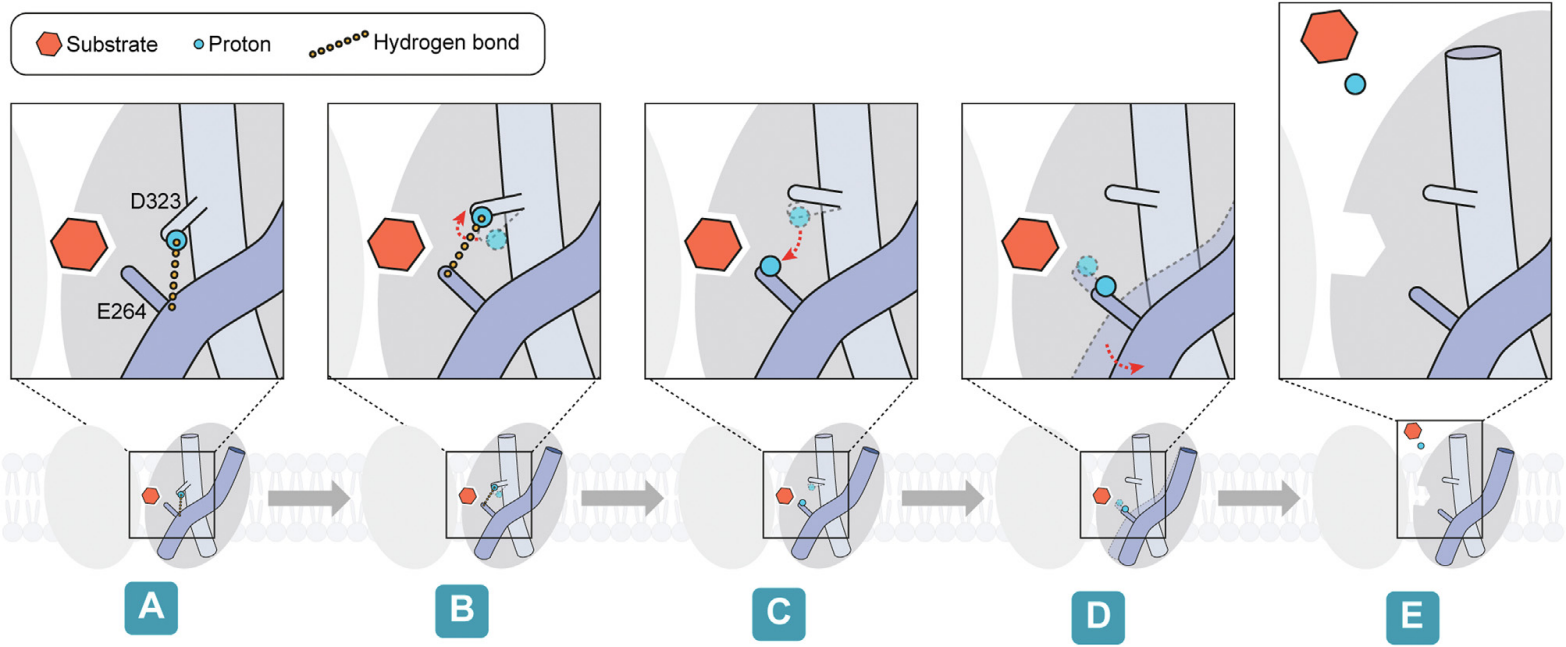
**The mechanism of substrate release in the NHS protein family.** NHS, nucleoside proton symporter.

This study provides a comprehensive analysis of the proton-coupled substrate release mechanism within the NHS protein family, focusing on the NupG protein as a model. Based on the analyses presented above, we propose a mechanism for proton-coupled substrate release using the NupG protein as a model for proteins belonging to the NHS subfamily. Initially, the Asp323 residue forms a proton bridge with the main chain oxygen atom of Glu264, while simultaneously binding the substrate in its protonated state (Fig. 8*A*). Subsequently, the side chain of Asp323 shifts, allowing the proton of its oxygen atom to interact with the side chain oxygen atom of Glu264 (Fig. 8*B*). Following this interaction, Asp323 transfers its proton to Glu264 (Fig. 8*C*). Once Asp323 is deprotonated, its side chain undergoes a significant flip, leading to the disruption of the TM10 helix and an outward shift of TM8 (Fig. 8*D*). This series of changes facilitates the release of the substrate and proton into the cell interior from the cavity (Fig. 8*E*).

Our structural analysis also extends to the YegT protein, another member of the NHS family. We found a modest 27% sequence identity between the YegT and NupG proteins, with structural alignment analysis revealing a high degree of conservation, particularly in the cavity region surrounding the protonation sites within the transmembrane domain. Moreover, MD simulations showed similar conformational changes at these sites and their corresponding transmembrane helices. Our observations suggest that the YegT and NupG proteins share common structural characteristics, in particular regarding their proton-coupled substrate release mechanisms, despite some differences likely exist in relation to substrate recognition and selection.

In addition, we identified a conserved GXXXD motif within transmembrane helices, which is distinct from the previously reported GXXXDRXGRR (motif A) that is predominantly found in the cytoplasmic loops L2-L3 or L8-L9 of the MFS protein family (13). While the presence of the transmembrane GXXXD motif has not been previously reported within the MFS family, similar sequences have been reported in transporter proteins belonging to other protein families, such as the human multidrug transporter Ptch1 of the RND family (15). Structural analysis of Ptch1 within the RND family shows that the D residue in this motif is able to form hydrogen bonds with carbonyl oxygen atoms located in the main chain (16, 17, 18), suggesting a potential protonated state for this D residue. The helix stabilized by the GXXXD motif is unstable, as indicated by the varying observed distances between the carbonyl oxygen atom of the G residue and the main chain nitrogen atom of the D residue in different protonation states (Fig. S4). Accordingly, our findings suggest that the GXXXD motif influences local conformations by affecting the stability of the alpha helix. Furthermore, previous biochemical studies showed that this motif functions as a proton relay closely linked to the transport activity of proteins belonging to the RND family (19). Given the localization of GXXXD motifs within the substrate-binding cavities of both NHS and RND families, along with structural data indicating D protonation, these observations highlight the critical role of the GXXXD motif in regulating substrate transport processes in the NHS family.

In summary, this study provides a comprehensive analysis of the structural characteristics of the NHS family members YegT and NupG through the implementation of crystallography and MD simulations. Despite the modest sequence identity uncovered between the YegT and NupG proteins, our findings reveal a high degree of structural conservation surrounding the protonation sites within the cavity region, which suggests potential similarities in their proton-coupled substrate release mechanisms. The identification of the GXXXD motif enhances our understanding of proton transfer roles in substrate transport within the MFS protein family, providing valuable insights into the functions of other members of this family. Future studies should focus on exploring the protonation dynamics of the GXXXD motif in various environments and validating its impact on protein function. Additionally, comparative studies with other family members could uncover novel regulatory mechanisms and drug targets, potentially serving as a foundation for developing innovative therapeutic strategies.

## Experimental procedures

### Protein purification

The genes encoding YegT and NupG were subcloned into the pET21b vector and subsequently transformed into BL21DE3 in *Escherichia coli*. Once the absorbanceat 600 nm reached approximately 1.0, protein overexpression was induced by adding 0.2 mM IPTG and incubating overnight at 18 °C. The cells were then harvested and resuspended in a buffer containing 25 mM Mes (pH 6.0) and 150 mM NaCl. Cells lysis was achieved using a high-pressure homogenizer set to 800 bar. The lysate was mixed with 2% (w/v) dodecyl β-D-maltoside (DDM, Anatrace) and incubated at 4 °C for 2 h. After centrifugation at 18,000 rpm for 40 min, the supernatant was collected and applied to a Ni-NTA affinity column (Qiagen) for purification. The column was washed with a buffer containing 25 mM Mes (pH 6.0), 150 mM NaCl, 25 mM imidazole, and 0.02% DDM. Proteins were then eluted with a buffer comprising 25 mM Mes (pH 6.0), 150 mM NaCl, 300 mM imidazole, and 0.02% DDM.

Further purification was carried out using size-exclusion chromatography with a Superdex 200 10/300 increase column (GE HealthCare). For proteins intended for ITC binding studies, the elution buffer was maintained as 25 mM Mes (pH 6.0), 150 mM NaCl, and 0.02% DDM. For crystallization preparation, the proteins were treated with a buffer containing 25 mM Mes (pH 6.0), 150 mM NaCl, and 0.4% n-octyl β-D-glucoside (NG). The peak fractions were collected and concentrated to approximately 30 mg/ml for use in subsequent crystallization experiments.

#### Crystallization

YegT was crystallized using the lipidic cubic phase method. The protein, at a concentration of 30 mg/ml, was mixed with monoolein in a 1:1.5 ratio (w/w). The mixture was thoroughly combined and spotted onto a special sandwich glass plate in 50 nl drops. The plate was then covered with 200 nl of the crystallization solution and sealed tightly. The setup was incubated at 20 °C in a temperature-controlled environment. Crystals typically began to form within approximately 3 days. The crystallization conditions for YegT were 0.1 M Mes (pH 5.8), 0. Li_2_SO_4_, 0.1 M glycine, and 26% PEG 550 MME. Once crystals were obtained, they were carefully harvested and rapidly cryocooled in liquid nitrogen to preserve the crystal structure.

#### Structure determination

Diffraction data were collected at the BL18U1 beamline of the Shanghai Synchrotron Radiation Facility and processed using XDS (20). The data were merged and scaled with Aimless from the CCP4 suite (https://www.ccp4.ac.uk/download/index.php#os=linux) (21). The structure of YegT was determined by molecular replacement with Phenix Phaser (https://phenix-online.org/download), using the NupG structure as the initial model (22, 23). The model was iteratively refined and built with Phenix Refinement (https://phenix-online.org/download) and Coot (https://www2.mrc-lmb.cam.ac.uk/personal/pemsley/coot/), leading to the final structure (23, 24). The data collection and refinement statistics are summarized in Table S1.

#### ITC experiments

Nucleotide binding to NupG was measured at 25 °C using a MicroCal iTC200. The purified NupG protein was concentrated to 40 to 60 μM for the ITC experiments. Uridine nucleoside was prepared at a concentration of 5 mM in the same buffer used for protein purification, which contained 25 mM Mes (pH 6.0), 150 mM NaCl, and 0.02% DDM. All experiments were performed in triplicate, and a representative result is presented.

#### MD simulations

SWISS-MODEL was first used to repair missing amino acid residues and side chains in the crystal structures of YegT and NupG with amino acid numbering ranging from 1 to 396 and 1 to 400, respectively. The structures were embedded in POPE lipids, and the protein-membrane system was solubilized in TIP3 water using CHARMM-GUI (25). Sodium and chloride ions were added to neutralize the system and achieve a final ion concentration of 0.15 M. The simulation box, measuring 106 Å × 106 Å × 103 Å, was subjected to periodic boundary conditions. The CHARMM36 force field parameters were used for proteins, lipids, ions, and solvent water (26). Energy minimization of the entire molecular system was performed using the steepest descent method until the maximum energy dropped below 1000 kJ/mol·nm. The system was then heated from 0 K to 100 K over 12.5 ps under the NVT ensemble, followed by heating to 310 K over 125 ps under the NPT ensemble (constant temperature and pressure). During equilibration, constraints were initially applied to the protein–ligand complex and lipid headgroups. The system was further equilibrated under the NPT ensemble at 1 bar pressure and 310 K temperature, gradually reducing the restraint force over six successive equilibration phases of 2.0 ns each. Final MD simulations were conducted for 200 ns under the NPT ensemble at 310K temperature and 1 bar pressure. The particle mesh Ewald method was used to calculate electrostatic interactions (19), and the LINCS algorithm constrained bond lengths involving hydrogen atoms (27). All MD simulations were performed using GROMACS software (https://manual.gromacs.org/) (28). Trajectory snapshots were saved every 40 ps, and simulation data were visualized and analyzed using Visual Molecular Dynamics (https://www.ks.uiuc.edu/Research/vmd/) and GROMACS software.

## Data availability

The atomic coordinates have been deposited in the Protein Data Bank (PDB accession code: 8ZOJ). All other data are contained within the article.

## Supporting information

This article contains supporting information.

## Conflict of interest

The authors declare that they have no conflicts of interest with the contents of this article.

## Supporting information

Supplyment material
