# Supplementary material for: Mechanistic insights into proton-coupled substrate translocation of nucleoside proton symporters
Source: J Biol Chem. 2025 Feb 25;301(4):108357. doi: 10.1016/j.jbc.2025.108357 (PMC11979927; doi:10.1016/j.jbc.2025.108357)
Supplement: Supplyment material [file mmc1.docx]

Table S1 The data collection and refinement statistics

| Data | YegT |
| --- | --- |
| Integration Package | XDS |
| Beamlines | BL18U1 |
| Space Group | P1 |
| Unit Cell (Å) | 42.8, 58.2, 105.1 |
| Unit Cell (°) | 77.8, 81.3, 68.5 |
| Wavelength (Å) | 0.9793 |
| Resolution (Å) | 50-2.9 |
| Rmerge (%) | 11.3 (92.9) |
| CC1/2 | 99.6 (67.3) |
| I/sigma | 7.95 (1.33) |
| Completeness (%) | 98.1(96.9) |
| Number of measured reflections | 68950 |
| Number of unique reflections | 19703 |
| Redundancy | 3.50 (3.45) |
| Rwork / Rfree (%) | 24.03/26.12 |
| No. atoms |  |
| Protein | 6177 |
| Others | 186 |
| Average B value (Å2 ) |  |
| Protein | 71.58 |
| Others | 68.81 |
| R.m.s. deviations |  |
| Bonds (Å) | 0.01 |
| Angle (°) | 1.35 |
| Ramachandran plot statistics (%) |  |
| Most favorable | 96.17 |
| allowed | 3.83 |
| Disallowed | 0 |

Table S1 ITC results summary

| Protein | Substrates | N=3 | | | Mean±SD ($\mu$M) |
| --- | --- | --- | --- | --- | --- |
| WT | Uridine | 287 | 261 | 243 | 263.67±22.12 |
| D323S |  | 58.4 | 45.7 | 52.3 | 52.13±6.35 |
| D323E |  | UD | UD | UD | UD |
| E264D |  | UD | UD | UD | UD |
| E264Q |  | UD | UD | UD | UD |


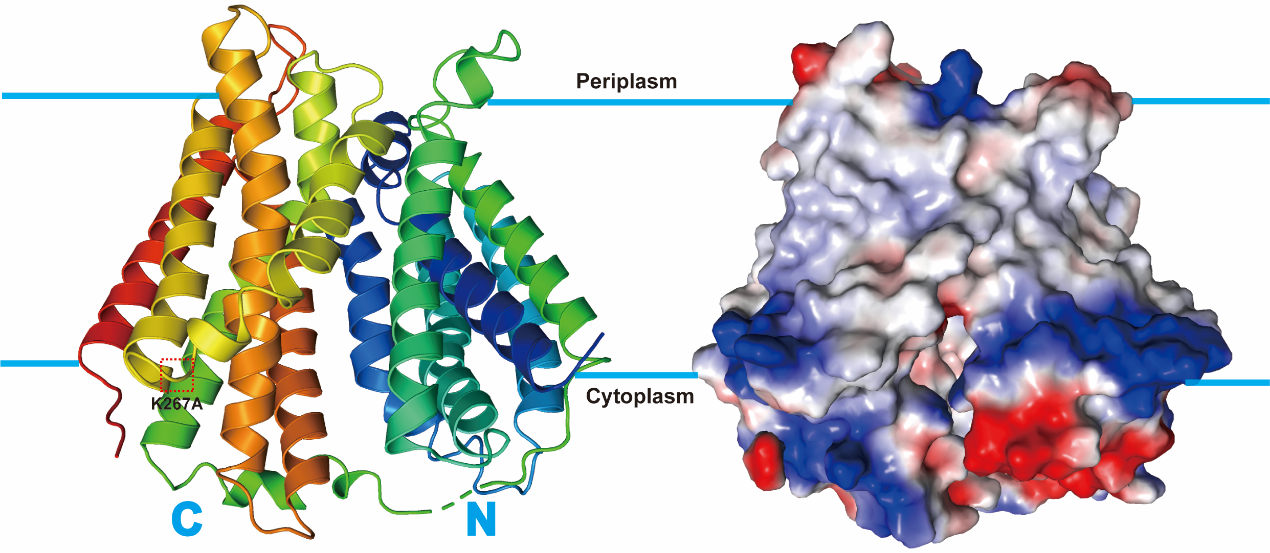


**Figure S1. The crystal structure of YegT_K267A_ (left) along with the map of its surface potential (right).**

**
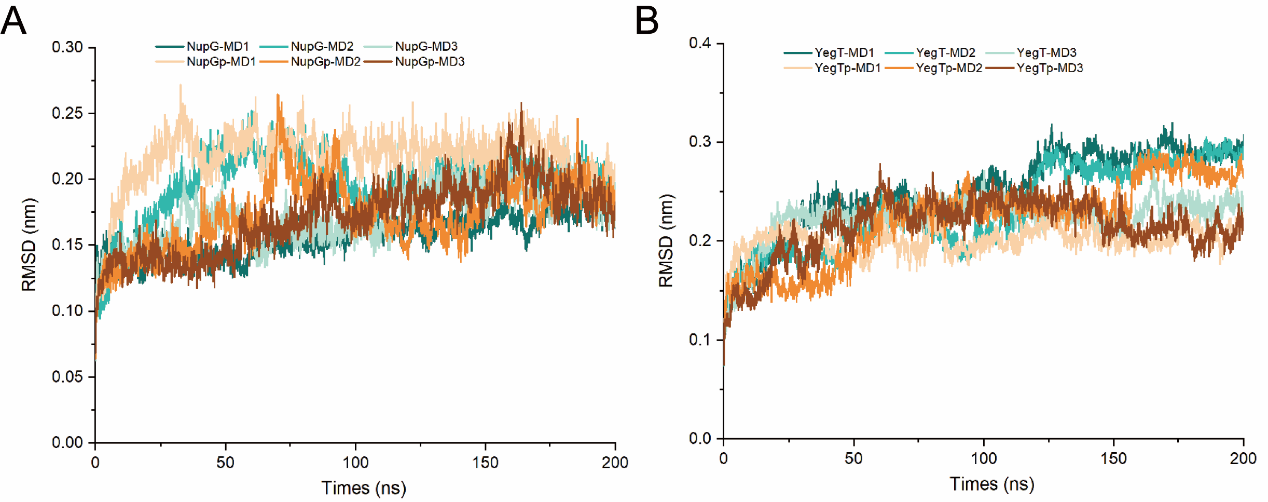
**

**Figure S2 Changes in the Root Mean Square Deviation (RMSD) of Ca atoms in the NupG and YegT proteins at different protonation states across a 200-nanosecond interval.** NupG-MD1, NupG-MD2, and NupG-MD3 refer to three independent simulations of NupG in a deprotonated state. NupGp-MD1, NupGp-MD2, and NupGp-MD3 refer to three independent simulations of NupG in a protonated state.

**
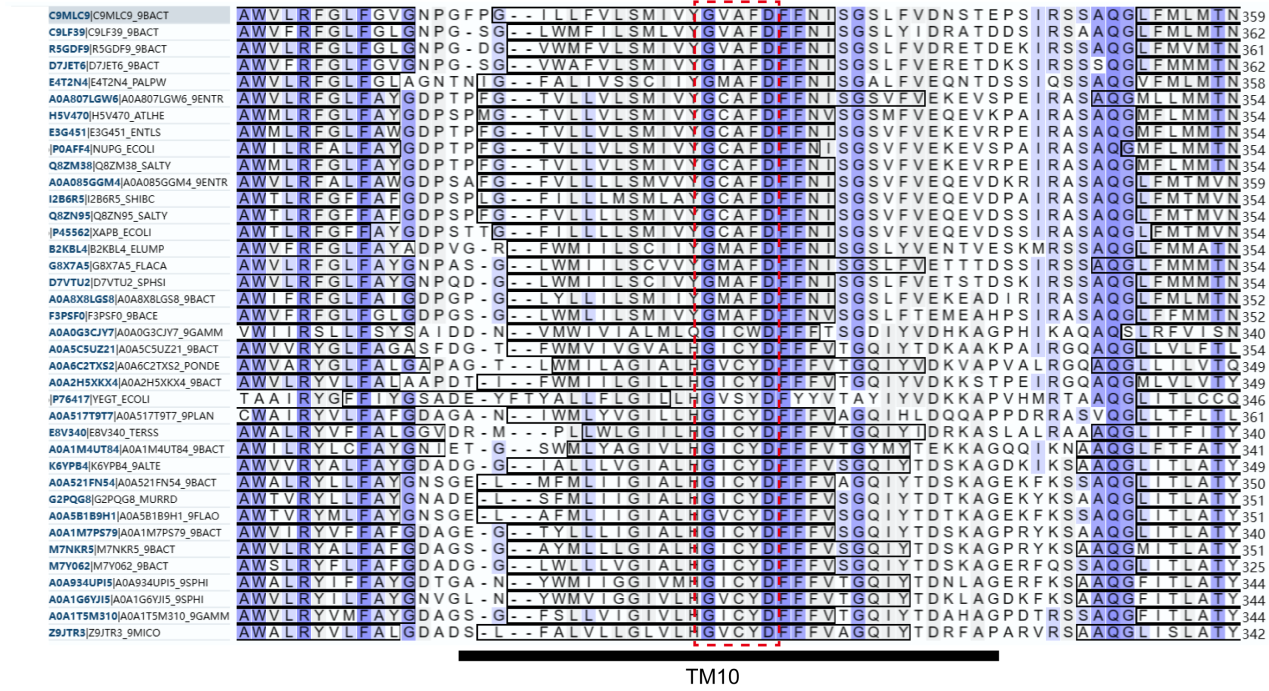
**

**Figure S3. Sequence alignment of members of the NHS protein subfamily in various species. The data was sourced from the Uniprot database.**

**
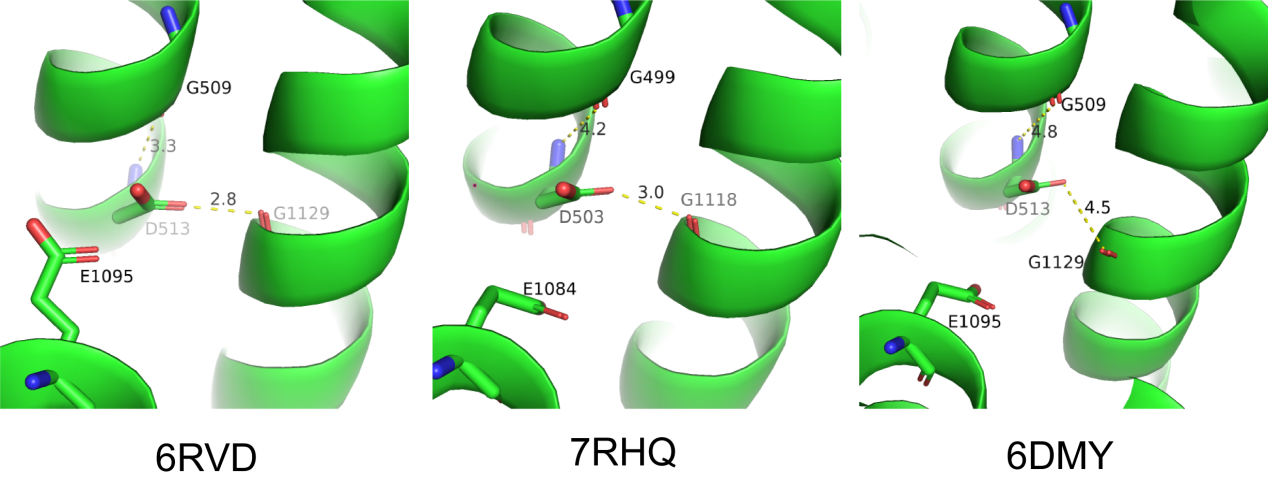
**

**Figure S4. The GXXXD motif in the Ptch1 protein belonging to the RND family (1-3).**

**References**

1. Rudolf, A. F., Kinnebrew, M., Kowatsch, C., Ansell, T. B., El Omari, K., Bishop, B. *et al.* (2019) The morphogen Sonic hedgehog inhibits its receptor Patched by a pincer grasp mechanism Nat Chem Biol **15**, 975-982 10.1038/s41589-019-0370-y

2. Huang, P., Wierbowski, B. M., Lian, T., Chan, C., García-Linares, S., Jiang, J. *et al.* (2022) Structural basis for catalyzed assembly of the Sonic hedgehog-Patched1 signaling complex Dev Cell **57**, 670-685.e678 10.1016/j.devcel.2022.02.008

3. Gong, X., Qian, H., Cao, P., Zhao, X., Zhou, Q., Lei, J. *et al.* (2018) Structural basis for the recognition of Sonic Hedgehog by human Patched1 Science **361**, 10.1126/science.aas8935
